# New Genes Tied to Endocrine, Metabolic, and Dietary Regulation of Lifespan from a *Caenorhabditis elegans* Genomic RNAi Screen

**DOI:** 10.1371/journal.pgen.0010017

**Published:** 2005-07-25

**Authors:** Malene Hansen, Ao-Lin Hsu, Andrew Dillin, Cynthia Kenyon

**Affiliations:** Department of Biochemistry and Biophysics, University of California, San Francisco, California, United States of America; Stanford University School of Medicine, United States of America

## Abstract

Most of our knowledge about the regulation of aging comes from mutants originally isolated for other phenotypes. To ask whether our current view of aging has been affected by selection bias, and to deepen our understanding of known longevity pathways, we screened a genomic *Caenorhabditis elegans* RNAi library for clones that extend lifespan*.* We identified 23 new longevity genes affecting signal transduction, the stress response, gene expression, and metabolism and assigned these genes to specific longevity pathways. Our most important findings are (i) that dietary restriction extends *C. elegans'* lifespan by down-regulating expression of key genes, including a gene required for methylation of many macromolecules, (ii) that integrin signaling is likely to play a general, evolutionarily conserved role in lifespan regulation, and (iii) that specific lipophilic hormones may influence lifespan in a DAF-16/FOXO-dependent fashion. Surprisingly, of the new genes that have conserved sequence domains, only one could not be associated with a known longevity pathway. Thus, our current view of the genetics of aging has probably not been distorted substantially by selection bias.

## Synopsis

Lifespan in *C. elegans* is influenced by several genetic pathways and processes; a great deal of the information about this regulation of aging comes from genetic mutants originally identified because of other phenotypes. Therefore, to ask whether the current view of the genetics of aging has been significantly affected by selection bias, and to deepen the understanding of known longevity pathways, Hansen et al. screened a genome-wide RNAi library for bacterial clones that extend lifespan when fed to the nematode *Caenorhabditis elegans.*

The investigators identified 23 new longevity genes affecting signal transduction, the stress response, gene expression, and metabolism and assigned these genes to specific longevity pathways. Their most important findings were (i) that dietary restriction extended *C. elegans'* lifespan by down-regulating expression of key genes, including a gene required for methylation of many macromolecules, (ii) that integrin signaling is likely to play a general, evolutionarily conserved role in lifespan regulation, and (iii) that specific lipophilic hormones may influence lifespan through the conserved insulin/IGF-1 signaling pathway.

Surprisingly, the authors found that of the new genes that have conserved sequence domains, only one could not be associated with a known longevity pathway. Thus, the current view of the genetics of aging has probably not been distorted substantially by selection bias. The authors expect the further study of these genes to provide valuable information about the mechanisms of aging, not only in *C. elegans* but also in higher organisms.

## Introduction

A number of mutations and environmental conditions can extend the lifespan of *Caenorhabditis elegans.* Although the underlying mechanisms are not fully understood, these perturbations appear to affect at least three distinct regulatory systems. The first is the insulin/IGF-1/FOXO system [1,56]. Inhibiting insulin/IGF-1 signaling can double the lifespan of the animal [54]. This lifespan extension requires the FOXO transcription factor DAF-16 [54], which, in turn, influences the expression of a diverse set of downstream antioxidant, metabolic, chaperone, antimicrobial, and novel genes that act in a cumulative way to influence lifespan [2,3]. The process of autophagy, which is increased in these long-lived mutants, may also make an important contribution [4]. In addition to DAF-16, the heat-shock transcription factor HSF-1 [5–7] as well as AMP kinase [8] are required for the lifespan extension of insulin/IGF-1 pathway mutants. The insulin/IGF-1/FOXO pathway acts exclusively during adulthood to influence aging [9] and appears to be regulated by sensory cues [10,11]. The reproductive system also affects insulin/IGF-1 signaling [12]. Killing the germline increases lifespan, and this lifespan increase requires the presence of the somatic gonad [12]. Germline ablation is thought to extend lifespan by activating a steroid hormone pathway and DAF-16/FOXO, and the somatic gonad appears to exert a counterbalancing influence on lifespan by affecting the activity of the insulin/IGF-1 pathway [12,13]. Finally, other regulators, such as the *sir-2* deacetylase and Jun kinase, can also increase lifespan in a *daf-16-*dependent fashion [14–17].

Dietary restriction (DR) (i.e., caloric restriction) also extends the lifespan of *C. elegans.* How this occurs is not clear. This lifespan extension does not require DAF-16 activity, suggesting that it is distinct from the insulin/IGF-1 signaling pathway [18,19] and the *sir-2* histone deacetylase [14]. So far, only one gene, the ubiquinone biosynthetic gene *clk-1,* has been implicated in the response to DR in *C. elegans* [18].

Third, inhibition of mitochondrial respiration or ATP synthesis increases lifespan [20–22]. Like the lifespan extension produced by DR, this lifespan extension is *daf-16-*independent. However, DR extends lifespan when it is initiated during adulthood, whereas respiratory-chain inhibition does not [21]. Thus, these two types of perturbations may act in different ways to increase lifespan.

In an effort to learn whether additional pathways might influence aging in *C. elegans,* and to identify additional genes in known pathways, we screened for enhanced longevity using a genomic RNA interference (RNAi) bacterial-feeding library that covers ~87% of the *C. elegans* open reading frames (total of 16,757) [23,24]. In our screen, we did not expect to identify all of the genes whose normal functions shorten lifespan. In previous Chromosome I RNAi screens, both the Ruvkun lab and our lab identified a number of mitochondrial RNAi clones that increased lifespan, but we identified only one gene in common [21,22] (legend in Table 1). Likewise, the Plasterk group found a high level of false negatives in each of their two RNAi screens [25]. In addition, we would not expect to find functionally redundant genes, genes with essential roles in other biological processes, or neuronal genes (since neurons are refractory to RNAi [24,26,27]). Nevertheless, since most biological pathways involve many genes, we hoped to identify genes that function in most or all of the pathways that influence lifespan.

**Table 1:**
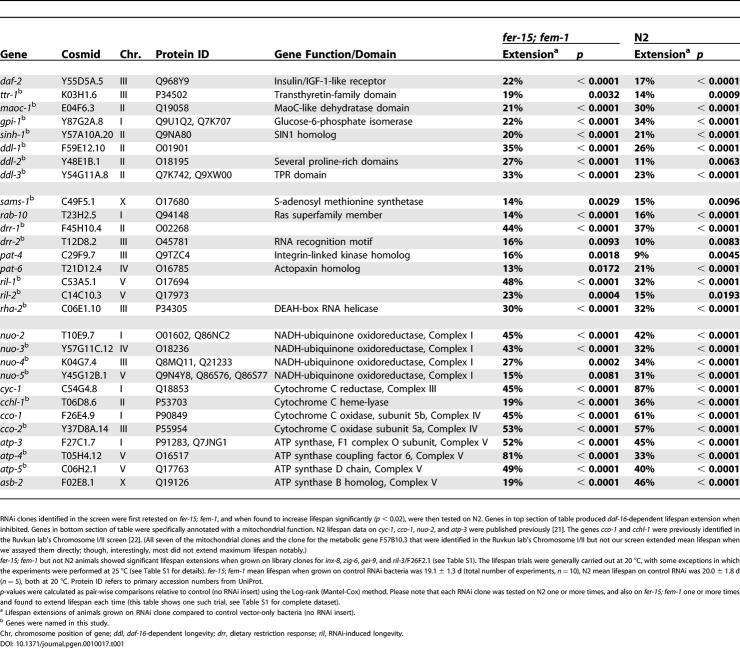
Mean Lifespan Extensions Observed in First Retests of *fer-15(b26); fem-1(hc17)* and of N2 Worms Grown on Long-Lived Candidate RNAi Clones

RNAi clones identified in the screen were first retested on *fer-15; fem-1*, and when found to increase lifespan significantly *(p* < 0.02), were then tested on N2. Genes in top section of table produced *daf-16*-dependent lifespan extension when inhibited. Genes in bottom section of table were specifically annotated with a mitochondrial function. N2 lifespan data on *cyc-1*, *cco-1*, *nuo-2*, and *atp-3* were published previously [21]. The genes *cco-1* and *cchl-1* were previously identified in the Ruvkun lab's Chromosome I/II screen [22]. (All seven of the mitochondrial clones and the clone for the metabolic gene F57B10.3 that were identified in the Ruvkun lab's Chromosome I/II but not our screen extended mean lifespan when we assayed them directly; though, interestingly, most did not extend maximum lifespan notably.)

*fer-15; fem-1* but not N2 animals showed significant lifespan extensions when grown on library clones for *inx-8*, *zig-6*, *gei-9*, and *ril-3*/F26F2.1 (see Table S1). The lifespan trials were generally carried out at 20 °C, with some exceptions in which the experiments were performed at 25 °C (see Table S1 for details). *fer-15*; *fem-1* mean lifespan when grown on control RNAi bacteria was 19.1 ± 1.3 d (total number of experiments, *n* = 10), N2 mean lifespan on control RNAi was 20.0 ± 1.8 d (*n* = 5), both at 20 °C. Protein ID refers to primary accession numbers from UniProt.

*p*-values were calculated as pair-wise comparisons relative to control (no RNAi insert) using the Log-rank (Mantel-Cox) method. Please note that each RNAi clone was tested on N2 one or more times, and also on *fer-15; fem-1* one or more times and found to extend lifespan each time (this table shows one such trial, see Table S1 for complete dataset).

^a^ Lifespan extensions of animals grown on RNAi clone compared to control vector-only bacteria (no RNAi insert).

^b^ Genes were named in this study.

Chr, chromosome position of gene; *ddl*, *daf-16*-dependent longevity; *drr*, dietary restriction response; *ril*, RNAi-induced longevity.

## Results/Discussion

### RNAi Screen for Clones That Increase Longevity

To identify longevity genes, we cultured animals on RNAi bacteria from the time of hatching and then looked for plates containing live individuals at a time when age-matched controls were all dead (see Materials and Methods). We estimate that we screened 70% of the open reading frames of *C. elegans* (Figure S1). Of the 94 candidate clones retested, 29 caused a highly significant lifespan extension, ranging from ~10–90%. (Five of these clones were published previously in Chromosome I/II screens [21,22], Table 1). These clones were tested a third time, and found to produce similar lifespan extensions (Tables 1 and S1).

Surprisingly, we recovered only one known longevity gene, the insulin/IGF-1-like receptor gene *daf-2.* To better understand this finding, we tested the RNAi clones of several known longevity genes (Table S2). Of the ten known genes we considered, only five were represented in our RNAi library. RNAi clones for three of these genes, *daf-2,* *age-1,* and *akt-1* (but not *clk-1* or *glp-1*), extended lifespan significantly (Table S2), indicating that we had a significant level of false negatives in our screen. (False negatives can arise for many reasons, including small sample size at the time of scoring, or censoring because of plate contamination or progeny production; see Materials and Methods. In addition, clones that extend only mean lifespan but not maximal lifespan would qualify as false negatives in our screen.) Together, these findings suggest that although we identified many new longevity genes in our screen, more remain to be found.

Some of the RNAi clones we recovered had relatively small effects on lifespan. Unexpectedly, one of these was the *daf-2* library clone (Figure S2), which repeatedly lengthened lifespan ~20% (see Tables 1 and S1). In contrast, a *daf-2* RNAi clone we constructed using the same vector but a different *daf-2* insert doubled lifespan (data not shown, Figure S2). Thus for *daf-2,* one could say that the RNAi library contained a “weak allele.” Likewise, for the other genes we analyzed, it is possible that longer lifespans could be produced by further reductions in gene activity.

The 23 new genes affected a wide variety of processes, including signal transduction, gene expression/nucleic-acid metabolism, the stress response, glucose and amino-acid metabolism, mitochondrial function, and regulation of vesicle trafficking (Table 1). We assigned these genes names based on our functional analysis and DNA sequence information (Table 1).

### New Components of the Insulin/IGF-1 and Reproductive Pathways

The DAF-16/FOXO transcription factor is required for mutations in the insulin/IGF-1 pathway, or germline ablation, to extend lifespan [1]. We found that seven of the new RNAi clones (*ttr-1/*K03H1.6, *maoc-1/*E04F6.3, *gpi-1/*Y87G2A.8, *sinh-1/*Y57A10A.20, *ddl-1*/F59E12.10*, ddl-2/*Y48E1B.1, and *ddl-3/*Y54G11A.8), as well as the *daf-2/*Y55D5A.5 clone, failed, in multiple trials, to extend the lifespan of *daf-16(mu86)* null mutants (Table 2). Such a failure to extend lifespan could conceivably be due to sickness, but the animals appeared healthy. (This was the case for all the other animals we examined, unless stated otherwise.) Therefore, the simplest explanation is that the activity of *daf-16* is specifically required to extend the lifespan of animals treated with these RNAi clones. In principle, these “*daf-16*-dependent” genes could function in the insulin/IGF-1 pathway, the reproductive pathway, both pathways, or other DAF-16-dependent pathways.

**Table 2:**
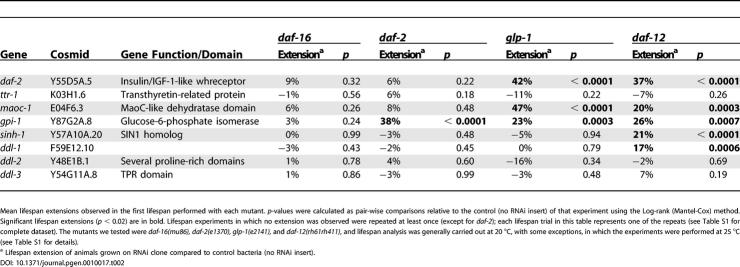
Genetic Epistasis Analysis of RNAi Clones Whose Effects Require DAF-16

Mean lifespan extensions observed in the first lifespan performed with each mutant. *p*-values were calculated as pair-wise comparisons relative to the control (no RNAi insert) of that experiment using the Log-rank (Mantel-Cox) method. Significant lifespan extensions (*p* < 0.02) are in bold. Lifespan experiments in which no extension was observed were repeated at least once (except for *daf-2*); each lifespan trial in this table represents one of the repeats (see Table S1 for complete dataset). The mutants we tested were *daf-16(mu86),* *daf-2(e1370), glp-1(e2141),* and *daf-12(rh61rh411),* and lifespan analysis was generally carried out at 20 °C, with some exceptions, in which the experiments were performed at 25 °C (see Table S1 for details).

^a^ Lifespan extension of animals grown on RNAi clone compared to control bacteria (no RNAi insert).

**Figure 1:**
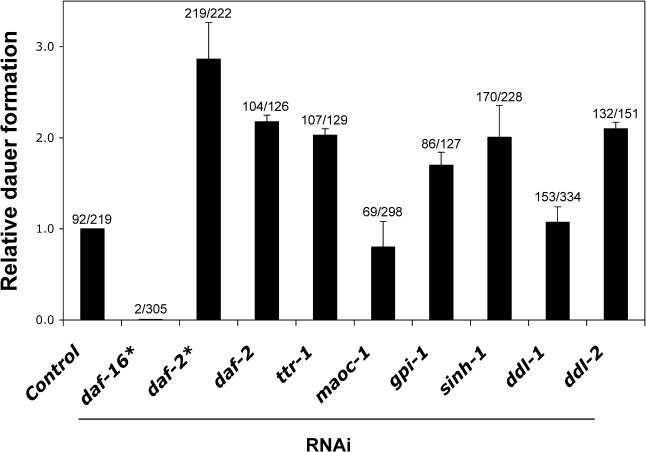
The Dauer-Constitutive Phenotype of *daf-2(e1370)* Is Enhanced by Many RNAi Clones That Extend Lifespan in a *daf-16-*Dependent Fashion Relative dauer formation of *daf-2(e1370)* animals grown at 22.5 °C on RNAi clones versus vector control is shown, average of two to three experiments. 30–50% of the animals on vector control become dauers at 22.5 °C. Total number of dauers/total number of animals observed is noted on top of bars. Error bars: ± SEM. ‘*', previously characterized RNAi clones [9] served as negative (*daf-16,* RNAi insert consists of first 1.2 kb cDNA) and positive (*daf-2,* RNAi insert consists of first 2.2 kb cDNA; see also Figure S2) controls for the dauer experiment. *daf-2(e1370)* worms grown on *ddl-3* RNAi gave rise to almost no progeny; therefore, this *daf-16*-dependent RNAi clone was not assayed.

New genes in the insulin/IGF-1 pathway are particularly interesting because their counterparts in humans could potentially play a role not only in longevity regulation but also in insulin/IGF-1-related metabolic diseases such as diabetes and cancer. To investigate whether the genes identified by these RNAi clones might be part of the insulin/IGF-1 pathway, we carried out two experiments. First, while not a conclusive test (since *daf-2* mutants are not null), we were curious to know whether the RNAi clones might be unable to further extend the lifespan of *daf-2(e1370)* mutants, as is the case with mutations in the downstream gene *age-1/*PI 3-kinase [28]. We found that this was the case for all but one of these clones (see below; Table 2)*.* In contrast, all but one of the *daf-16-*independent clones that we examined increased the lifespan of *e1370* mutants (see below; Table 3). We also asked whether these RNAi clones might affect a different process regulated by the insulin/IGF-1 pathway: dauer formation. Wild-type juveniles enter dauer, a pre-pubescent growth-arrested state, in response to food limitation. Strong *daf-2* mutants become dauers even in the presence of food, in a *daf-16-*dependent fashion. To assay dauer formation, we asked whether our *daf-16-*dependent RNAi clones enhanced the weak dauer-constitutive (Daf-c) phenotype of *daf-2(e1370)* mutants cultured at 22.5 °C. We found that four *daf-16*-dependent RNAi clones *(ttr-1,* *gpi-1,* *sinh-1,* and *ddl-2)* enhanced dauer formation to a significant extent, whereas two clones *(maoc-1* and *ddl-1)* did not (Figure 1). (*daf-2(e1370)* worms grown on *ddl-3* RNAi produced almost no progeny. Therefore, this *daf-16*-dependent RNAi clone was not assayed.) Because *ttr-1,* *gpi-1,* *sinh-1,* and *ddl-2* RNAi clones all enhanced dauer formation and their function was *daf-16*-dependent, these genes are likely to be part of the *daf-2* pathway.

**Table 3:**
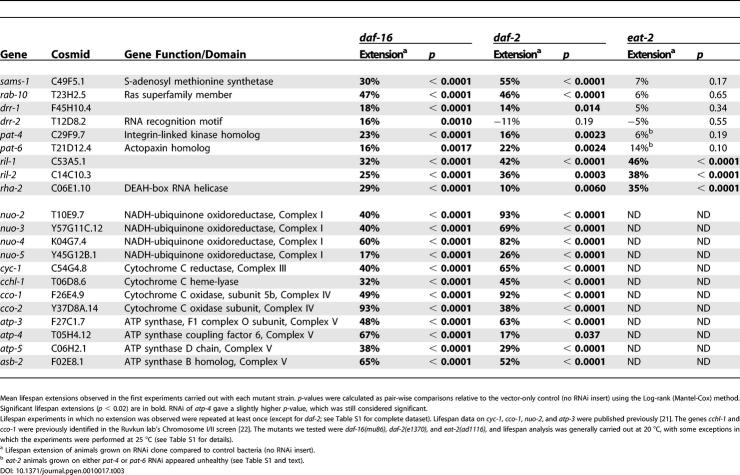
Genetic Epistasis Analysis of RNAi Clones Whose Effects Do Not Require DAF-16

Mean lifespan extensions observed in the first experiments carried out with each mutant strain. *p*-values were calculated as pair-wise comparisons relative to the vector-only control (no RNAi insert) using the Log-rank (Mantel-Cox) method. Significant lifespan extensions (*p* < 0.02) are in bold. RNAi of *atp-4* gave a slightly higher *p*-value, which was still considered significant.

Lifespan experiments in which no extension was observed were repeated at least once (except for *daf-2*; see Table S1 for complete dataset). Lifespan data on *cyc-1*, *cco-1*, *nuo-2*, and *atp-3* were published previously [21]. The genes *cchl-1* and *cco-1* were previously identified in the Ruvkun lab's Chromosome I/II screen [22]. The mutants we tested were *daf-16(mu86),* *daf-2(e1370),* and *eat-2(ad1116),* and lifespan analysis was generally carried out at 20 °C, with some exceptions in which the experiments were performed at 25 °C (see Table S1 for details).

^a^ Lifespan extension of animals grown on RNAi clone compared to control bacteria (no RNAi insert).

^b^ *eat-2* animals grown on either *pat-4* or *pat-6* RNAi appeared unhealthy (see Table S1 and text).

To test whether our *daf-16*-dependent RNAi clones were part of the germline pathway, we asked whether they could further extend the lifespans of *glp-1(e2141ts)* animals (which lack a germline when raised at high temperature [29]), and also mutants defective in the putative steroid receptor *daf-12* [12]. The germline pathway is distinct from the *daf-2* pathway in that it does not appear to affect dauer formation (see data in Materials and Methods). Consistent with this, our *daf-2* clone further extended the lifespan of *glp-1* mutants (see Table 2). As described below, five of our *daf-16*-dependent RNAi clones *(ttr-1, sinh-1, ddl-1, ddl-2,* and *ddl-3)* failed to extend the lifespan of *glp-1* mutants. In principle, these clones could simply prevent the development of the germline. However, this seems unlikely, because none of these RNAi clones reduced brood size (data not shown). Instead, we favor the interpretation that germline ablation extends lifespan, at least in part, by inhibiting the activities of these five genes. These and other *daf-16-*dependent genes will now be described in more detail.

One of the most interesting *daf-16-*dependent clones contains a transthyretin-family domain and the corresponding protein is thus predicted to be a member of the transthyretin-related protein family (*ttr-1,* for transthyretin-related protein). In vertebrates, transthyretin is one of three specific carrier proteins involved in the transport of both thyroid hormones and retinol. These carrier proteins might also play a role in regulating the uptake of the free circulating hormones in various tissues [30,31]. *C. elegans* TTR-1 is a member of a distinct but related protein family with a completely conserved predicted hydrophobic pocket [32], suggesting that it could function as a carrier of a lipophilic hormone(s) that influences lifespan. We found that *ttr-1* RNAi enhanced dauer formation (Figure 1) and failed to further extend the lifespan of *daf-2(e1370)* mutants (see Table 2), suggesting that it (and the putative lipophilic hormone[s] it binds) acts in the insulin/IGF-1 pathway either upstream of or in parallel to *daf-16.*

The *ttr-1* RNAi clone did not further extend the lifespan of *glp-1* mutants (see Table 2), suggesting that it also functions in the germline pathway to influence lifespan. The lifespan extension of germline-ablated animals requires the putative steroid hormone receptor DAF-12 [12], and we found that *ttr-1* was unable to extend the lifespan of DAF-12 mutants. Thus, one plausible model is that TTR-1 binds to and limits the availability of a ligand that activates DAF-12.

Another *daf-16-*dependent RNAi clone, *maoc-1,* encodes a protein containing a MaoC-like dehydratase domain, which is also found in enzymes such as human type IV estradiol 17-β-dehydrogenase and in the fatty acid synthase β subunit. Therefore, *maoc-1* could act in the synthesis of a lipophilic hormone that influences lifespan in a *daf-16-*dependent fashion. This clone did not further extend the lifespan of *daf-2(e1370)* mutants (see Table 2), suggesting that the gene acts in the insulin/IGF-1 pathway; however, it failed to enhance the Daf-c phenotype (Figure 1). Furthermore, the clone enhanced the longevity of *glp-1* (germline-defective) mutants (see Table 2), arguing that it does not extend lifespan by affecting germline signaling. The clone also increased the lifespan of *daf-12-*null mutants, so it is unlikely to synthesize a DAF-12 ligand. Since this clone acts differently from *ttr-1* to affect lifespan, these findings raise the possibility that multiple lipophilic hormones influence lifespan in a *daf-16-*dependent fashion.

The third *daf-16-*dependent gene, *gpi-1,* encodes a glucose-6-phosphate isomerase/neuroleukin homolog. This RNAi clone enhanced dauer formation (Figure 1) and extended the lifespan of *glp-1* and *daf-12* mutants (see Table 2). Thus, it probably functions in the insulin/IGF-1 pathway, but not in the reproductive pathway to affect lifespan. In mammals, glucose-6-phosphate isomerase functions in glycolysis and also as a secreted neuronal growth factor. We favor the possibility that glycolysis influences aging, because inhibition of another glycolysis gene, phosphoglycerate mutase, also extends lifespan [22]. One possibility is that this RNAi clone extends lifespan by reducing ATP levels, which in turn inhibits the release of insulin-like DAF-2 ligands—analogous to the effect of ATP on insulin release in mammals [33].

The fourth *daf-16-*dependent gene, *sinh-1,* encodes a homolog of *Schizosaccharomyces pombe* *SIN1,* stress-activated MAP kinase interacting protein 1 [34], which functions in the response to DNA damage. In *S.* *pombe, SIN1* inactivation increases sensitivity to some environmental stresses, such as high temperature and osmotic stress, but not to oxidative stress [34]. Unexpectedly, we found that *sinh-1* RNAi significantly increased both thermotolerance (mean lifespan of wild-type worms at 35 °C: control 16.0 h; *sinh-1(RNAi)* 20.6 h; *p* < 0.0001) and the resistance to oxidative stress (mean lifespan of wild-type worms treated with paraquat (free-radical generator): control 4.4 h; *sinh-1(RNAi)* 7.1 h; *p* < 0.0001). Thus, in *C. elegans* (and perhaps other multicellular organisms as well), this gene appears to prevent, rather than promote, stress resistance.

*sinh-1* RNAi enhanced dauer formation (Figure 1) and failed to further extend the longevity of *daf-2(e1370)* mutants (see Table 2), suggesting that it acts in the insulin/IGF-1 pathway. *sinh-1* RNAi also failed to extend the longevity of *glp-1* mutants, suggesting that *sinh-1* functions in the germline pathway (see Table 2). Interestingly, *sinh-1* RNAi did extend the lifespan of *daf-12/*steroid-receptor mutants (see Table 2). The regulatory relationship between DAF-16 and DAF-12 is not known. Thus, one possible explanation is that in response to germline ablation, DAF-12 activates DAF-16/FOXO by inhibiting the activity of SINH-1. Alternatively, germline cells might regulate the activities of DAF-16/FOXO and DAF-12 in parallel, and *sinh-1* might act only in the pathway that regulates DAF-16/FOXO.

The protein encoded by the fifth *daf-16-*dependent RNAi clone, *ddl-1* (for *daf-16*-dependent longevity), has been reported to interact with heat-shock factor binding protein (HSB-1) [35], a negative regulator of *C. elegans* heat-shock transcription factor (HSF-1) activity [36]. In *C. elegans,* HSF-1 promotes longevity [5–7] and is required along with *daf-16/*FOXO for the increased lifespan of *daf-2* receptor mutants [6,7]. Thus, wild-type *ddl-1* might inhibit longevity by reducing the activity of HSF-1. *ddl-1* RNAi did not further extend the lifespan of *daf-2(e1370)* mutants (see Table 2), consistent with a role in the insulin/IGF-1 pathway, but it did not enhance dauer formation (Figure 1).

HSF-1 is also required for the longevity of animals lacking a germline (*hsf-1(RNAi)* mean lifespan, 10.4 d at 20 °C, n=53; *glp-1(e2141); hsf-1(RNAi)* mean lifespan 11.0 d, n=60; *p* = 0.25). *ddl-1* RNAi did not extend the lifespan of *glp-1* mutants, suggesting that it, too, functions in the germline pathway. Interestingly, like *sinh-1* RNAi*, ddl-1* RNAi was able to extend the lifespans of *daf-12* mutants (see Table 2). This finding suggests that *ddl-1* and *sinh-1,* which was also implicated in the stress response, act in the same process to affect longevity.

The sixth *daf-16*-dependent clone, *ddl-2,* encodes a protein that has been reported to interact with DDL-1 [35]. This RNAi clone enhanced dauer formation (Figure 1) and failed to increase the longevity of *daf-2(e1370)* mutants (see Table 2), consistent with a role in the insulin/IGF-1 pathway. Like *ddl-1* RNAi, *ddl-2* RNAi failed to increase the lifespan of germline-deficient animals (see Table 2), suggesting that it, too, functions in the germline pathway. However, unlike *ddl-1* RNAi, in several experiments, this clone failed to extend the lifespan of *daf-12* mutants (see Table 2). Assuming that DDL-1 and DDL-2 do interact, this suggests that they function in a complex fashion to influence lifespan.

The final *daf-16-*dependent clone, *(ddl-3),* is predicted to have a tetratrico-peptide-repeat (TPR) protein interaction motif. This clone failed to increase the longevity of *daf-2* mutants (see Table 2), suggesting that *ddl-3* acts in the insulin/IGF-1 pathway. It also failed to further extend the lifespan of *glp-1/*germline-defective and *daf-12/*steroid-receptor mutants (see Table 2), suggesting that DDL-3 also acts in the germline pathway, possibly as an adaptor protein in a signaling cascade that regulates DAF-16 and DAF-12.

### Mitochondrial RNAi Clones

Given the large number and large effects of Chromosome I/II respiratory-chain RNAi clones [21,22], we were not surprised to find that 12 of our RNAi clones encoded components of the mitochondrial respiratory chain. (Of these, four Chromosome I clones [*nuo-2*/T10E9.7, *cyc-1*/C54G4.8, *cco-1*/F26E4.9, and *atp-3*/F27C1.7] and one Chromosome II clone [*cchl-1*/T06D8.6] were published previously [21,22]. The seven new clones were *nuo-3*/Y57G11C.12, *nuo-4*/K04G7.4, *nuo-5*/Y45G12B.1, *cco-2*/Y37D8A.14, *atp-4*/T05H4.12, *atp-5*/C06H2.1, and *asb-2*/F02E8.1 [see Table 1].) Respiratory-chain RNAi clones are thought to affect a pathway that is independent of the insulin/IGF-1 pathway [21,22]. Consistent with this, we found that all of the new mitochondrial RNAi clones were able to extend the lifespan of *daf-2* mutants, and that their activities were *daf-16* independent (Table 3).

RNAi of respiratory-chain components decreases body size and slows movement and eating behavior (pumping) [21]. Interestingly, while lifespan and size/pumping rate are inversely correlated for most of the animals in this class, there were several exceptions (see Table S3). For example, we found that animals subjected to *nuo-5* (NADH-ubiquinone oxidoreductase) or *cchl-1* (cytochrome C heme-lyase) RNAi had almost normal body size (and, for *nuo-5,* also normal pumping rate) but substantial lifespan extensions (about 30%; see Table 1). Thus, the longevity of animals with reduced respiration is unlikely to be causally connected to their small size or reduced rate of pumping.

### Genes That May Be Involved in the Longevity Response to Dietary Restriction

Nine additional RNAi clones also increased the lifespan of *daf-16* mutants (Table 3). DR extends the lifespan of *C. elegans* in a *daf-16*-independent manner [18,19]. We therefore asked whether any of these clones might be involved in the response to DR. To do this, we asked whether they might fail to further extend the long lifespan of the *eat-2(ad1116)* mutant, which is a genetic model for DR [18,37]. We found that this was the case for four (*sams-1*/C49F5.1, *rab-10*/T23H2.5, *drr-1/*F45H10.4, and *drr-2*/T12D8.2) of the nine genes (Table 3). This was interesting because to date, the lifespan of all but one long-lived mutant examined, *clk-1,* can be extended further by mutations in *eat-2* [18].

**Figure 2:**
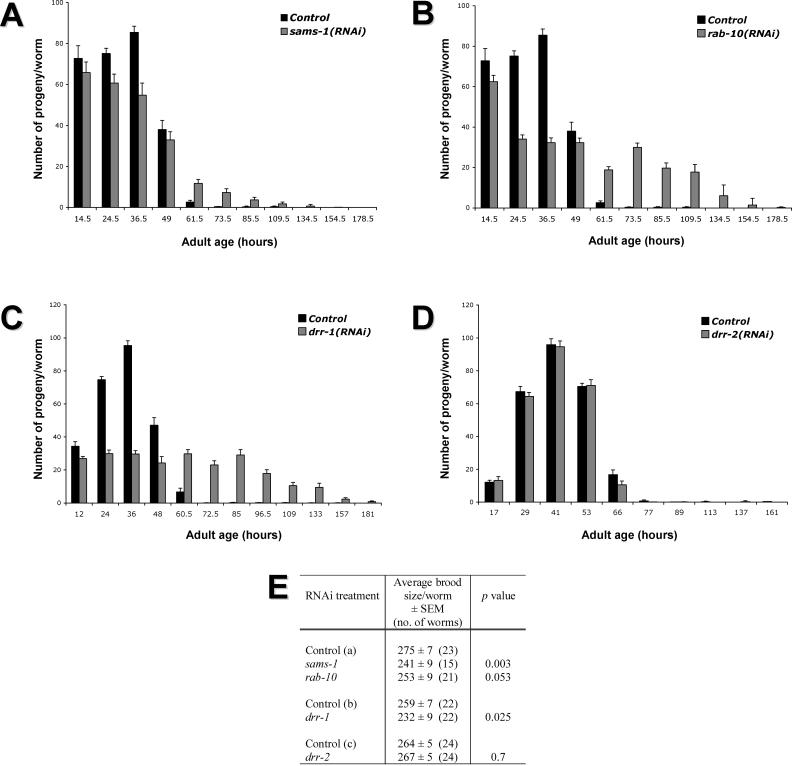
*sams-1,* *rab-10,* and *drr-1* RNAi Affect Reproduction Progeny profile of N2 animals grown on RNAi clones for (A) *sams-1,* (B) *rab-10,* (C) *drr-1,* and (D) *drr-2* (note that *drr-2* RNAi did not affect reproduction). Number of progeny per worm at each time interval is shown. Error bars: ± SEM. (E) Total brood size of N2 worms grown on RNAi clones for either *sams-1,* *rab-10, drr-1,* or *drr-2*. The number of progeny produced by each worm was calculated from the progeny profile data in (A)–(D) and averaged. The *p*-values were calculated relative to control of the experiment as Student's *t*-test.

**Figure 3:**
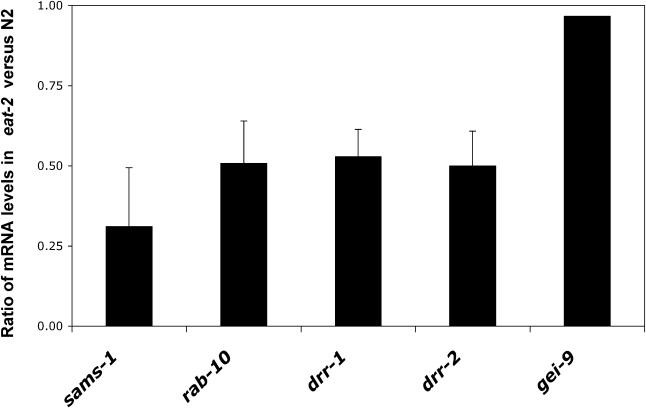
mRNA Levels of *sams-1,* *rab-10, drr-1,* and *drr-2* Are Reduced in *eat-2(ad1116)* Mutants Relative mRNA levels of *sams-1,* *rab-10, drr-1,* and *drr-2* in *eat-2(ad1116)* compared to N2 were measured by quantitative PCR, and average of four different sample sets are shown. The relative mRNA levels were normalized against the *act-1* (beta-actin) level in each sample. The RNAi clone for *gei-9* is shown as a control; this clone does not cause significant lifespan extension when fed to N2 or *eat-2* worms (Table S1, and data not shown). Error bars: ± SEM.

One of these genes, *sams-1,* encodes S-adenosyl methionine synthetase, a protein that functions as a universal methyl group donor in many biochemical reactions. Inhibition of this enzyme can affect methylation of histones, DNA, RNA, proteins, phospholipids, and other small molecules [38]. To further investigate the possibility that *C. elegans sams-1* plays a role in the response to DR, we asked whether this RNAi clone, like DR [39] (and D. Crawford and C. Kenyon, unpublished data) reduced brood size and delayed reproduction. We found that brood size was reduced and reproductive timing was slightly delayed (Figure 2). In addition, like DR animals, *sams-1* RNAi, worms were slender (see Table S1). The rate of pumping (eating) was not affected (data not shown), suggesting that this RNAi clone exerts its effects via changes in metabolism rather than changes in appetite. Together, these findings suggest that DR may extend lifespan, at least in part, by inhibiting the activity of *sams-1.* Consistent with this idea, we found that *sams-1* mRNA levels were reduced 3-fold in *eat-2* mutants (Figure 3). We therefore propose the model that DR may initiate a longevity response, at least in part, by triggering a regulated decrease in *sams-1* mRNA levels and, consequently, cellular S-adenosyl methionine levels. Reducing dietary methionine levels is known to increase the lifespan of mice [40] and rats by 40–45% [41–43]; however, whether methionine limitation and general DR activate the same longevity mechanisms is not known. Our findings suggest that this might be the case.

Another RNAi clone that failed to further extend the lifespan of *eat-2* mutants was *rab-10,* which encodes a Rab-like GTPase similar to those that regulate vesicle transport. Like *sams-1* RNAi, this clone did not affect pumping, but did reduce and delay reproduction (see Figure 2) and produced a slender appearance (see Table S1). The expression of this gene, too, was down-regulated in response to DR (2-fold; Figure 3). All of these properties were shared by *drr-1* (dietary restriction response), a gene encoding a novel protein with no obvious human homolog (see Table S1 and Figures 2 and 3).

The phenotype of the last DR clone, *drr-2,* differed from those of the other DR clones in that *drr-2(RNAi)* worms had a normal, well-fed appearance (see Table S1) and normal reproduction (see Figure 2). DRR-2 is a putative RNA-binding protein, suggesting that it plays a regulatory role in triggering the longevity response (but not the reproductive response) to DR. Expression of this gene was also reduced in *eat-2* mutants (2-fold, Figure 3), suggesting that, like the other genes we identified, down-regulation of this gene in response to DR somehow causes lifespan extension. In general, it was striking that expression of all four DR genes was reduced in *eat-2* mutants. This suggests that in *C. elegans,* DR elicits a concerted transcriptional (or mRNA turnover) response that can inhibit multiple lifespan-shortening gene activities and thereby extends lifespan.

We also asked whether the lifespan of *eat-2(ad1116)* mutants could be increased by respiratory-chain RNAi, and found that it could (D. Crawford and C. Kenyon, unpublished data). Since reducing respiratory chain activity during development is required for lifespan extension [21], whereas reducing food levels only during adulthood extends lifespan [44], it seems likely that mitochondrial respiratory chain components and DR do not extend lifespan in exactly the same way.

### Integrin Signaling is Likely to Play an Evolutionarily Conserved Role in Lifespan Limitation

Loss-of-function mutations in β-integrin *(myospheroid)* extend lifespan in *Drosophila* [45], and we found that RNAi clones of *pat-4/*C29F9.7, which encodes integrin-linked kinase, and *pat-6*/T21D12.4, which encodes actopaxin, a protein known to bind to integrin-linked kinase [46], increased lifespan in *C. elegans* (see Table 1)*.* Integrin signaling influences insulin-signaling pathways in mammals [47], yet the lifespan extension produced by these two clones was *daf-16* independent (Table 3). In addition, the lifespan of *daf-2(e1370)* mutants was extended when these animals were grown on either *pat-4* or *pat-6* RNAi (Table 3). Thus, integrin signaling may comprise a novel, conserved lifespan regulatory pathway, though it could potentially function in the insulin/IGF-1 pathway downstream of *daf-16—*which is known to act cell-non-autonomously [48]—to influence lifespan. (Inactivation of *pat-4* and *pat-6* impaired the health of *eat-2* mutants, making their relationship to DR difficult to interpret.) The finding that genes or pathways already known to influence the lifespan of another organism also affect *C. elegans'* lifespan is significant, as such ancient, evolutionarily conserved longevity pathways could potentially also influence human lifespan.

### Genes That Might Function in Novel Pathways to Influence Longevity

Using genetic epistasis analysis, we were able to associate most of our new genes with known aging-regulatory pathways or processes. However, the roles of three genes remained unclear. Two of these, *ril-1*/C53A5.1 and *ril-2*/C14C10.3 (RNAi-induced longevity), encoded novel proteins with no obvious homologs, whereas *rha-2*/C06E1.10 encoded a DEAH RNA helicase, suggesting that it regulates gene expression or nucleic-acid metabolism. All three RNAi clones extended the lifespan of both *daf-16* and *eat-2* mutants (Table 3), suggesting that they may function in a novel pathway or pathways to influence longevity.

### Summary

In this study, we identified many interesting new genes whose normal function is predicted to inhibit longevity. Although our screen did not reach saturation, an interesting picture emerged. Most of the genes we identified fell into one of three classes: genes that influence lifespan through DAF-16/FOXO (8/29), genes that influence respiration (12/29), and genes that appear to affect the response to DR (4/29). Two more genes affected integrin signaling, which was known to influence lifespan in flies. Like the insulin/IGF-1/FOXO system and the respiratory chain, most biological pathways and systems consist of many genes, and we failed to identify even one component of many such systems (e.g., the TGF-β signaling system). In fact, of the genes that had conserved sequence motifs, only one, the *rha-2* RNA helicase homolog, could not be linked to a known pathway. These findings are thought-provoking because until now, we have had no way of knowing whether the longevity pathways we know about represent only the tip of the iceberg. Our findings suggest that, in contrast, at most only one or a few other large multigenic systems influence lifespan in *C. elegans.* In other words, we may now be aware of most of the major biological pathways in *C. elegans* that, when inhibited, can produce large extensions in lifespan.

## Materials and Methods

### 

#### Strains.

All strains were maintained as described previously [49]. CF1037: *daf-16(mu86)I,* DA1116: *eat-2(ad1116)II,* CF1041: *daf-2(e1370)III,* CB4037: *glp-1(e2141)III,* MQ887: *isp-1(qm150)IV,* AA86: *daf-12(rh41rh411)X,* CF512: *fer-15(b26)II; fem-1(hc17)III*. All strains displayed similar Unc phenotypes when grown on *unc-52* RNAi bacteria (data not shown).

#### RNAi aging screen.

The systematic RNAi screen was carried out as described [23,24] with some modifications. Each RNAi bacteria colony was grown at 37 °C in LB with 10 μg/ml tetracycline and 50 μg/ml carbenicillin, and then seeded onto NG-carbenicillin plates supplemented with 100 μl 0.1 M IPTG. For our screen, we employed a sterile strain, CF512 (*fer-15(b26); fem-1(hc17)*) [5], to avoid transferring aging worms away from their progeny. Approximately 60 eggs of CF512 were added to the RNAi plates and allowed to develop to adults at 25 °C and kept at this temperature (Chromosome I, II, and first half of Chromosome X) or shifted to 20 °C for the rest of their life (Chromosome III, IV, V, and second half of Chromosome X). As a positive control, we used a previously described *daf-2* clone (pAD48, [9]), and as a negative control, we used the corresponding empty vector (pAD12, [9]). DAF-2 functions in both neural and non-neural tissues to influence lifespan [48,50,51], and *daf-2* RNAi has been shown previously to double the lifespan of the animal [9]. Developmental phenotypes were scored at d 1, and additional 100 μl 0.1 M IPTG was added on d 7. Viability of worms on each plate was scored at d 24 of adulthood (25 °C) or d 30 of adulthood (20 °C), at which time generally all worms on control plates were dead and 75–95% of worms grown on *daf-2* RNAi (pAD48) were still alive. RNAi appears to remain effective on older plates, since 30-d-old and newly seeded *unc-52* RNAi plates were equally effective at inducing an Unc phenotype (data not shown). Plates were censored due to contamination, progeny production, no bacterial growth, etc. (see Figure S1). We then carried out quantitative survival analysis using both CF512 and N2 with all of the bacterial strains that scored positively in the screen. Unexpectedly, RNAi knockdown of four of the genes we retested, *inx-8* (innexin-8), *ril-3* (F26F2.1), *zig-6* (protein with immunoglobulin domains), and *gei-9* (similarity to acyl-CoA dehydrogenase), significantly extended lifespan of CF512 but not N2 animals (see Table S1).

#### RNAi clone analysis.

The identity of all positive RNAi clones was verified by sequencing of inserts with an M13-forward primer, and, upon every start of a lifespan analysis, by PCR with T7 primers. About half of the genes we identified in our screen are located in operons. Therefore, we considered the possibility that the phenotypes we observed were influenced by RNAi knockdown of co-transcribed genes. This type of “intra-operonic inhibition,” though rare, has been observed [52,53]. However, none of these RNAi clones produced the phenotypes predicted for knockdown of other genes in the same operon (data not shown). All gene annotations were based on WormBase and/or WormPD, and protein accession numbers were from UniProt.

#### Lifespan analysis.

Lifespan analysis was conducted at 20 °C as described previously unless otherwise stated [10,54]. Eggs were added to plates seeded with RNAi bacteria, and animals were transferred approximately every week to newly seeded plates. At least 60 worms were used for each experiment. More animals were included in the analysis to ensure sufficient power when the lifespan extension of a particular RNAi clone was expected to be minimal. To reduce the chance of false negative results, all RNAi clones that failed to extend the lifespan of a particular mutant strain were retested at least once more with the same strain (except for *daf-2(e1370)*, see Table S1)*.* Because of the large number of lifespan experiments conducted, we did not always perform positive controls with CF512 or N2 animals exactly in parallel with each mutant we examined. However, these controls were running in overlapping time frames, where they consistently extended lifespan, generally (in 21 of 28 repeated experiments for N2/CF512 and in 43 of 57 repeated experiments when including data on all strains used) by magnitudes that did not differ by more than ten percentage points from one another (see Table S1). All positive RNAi clones extended lifespan in at least four independent trials, including analysis of all genetic mutants.

In all experiments, the pre-fertile period of adulthood was used as *t* = 0 for lifespan analysis. Strains were grown at 20 °C at optimal growth conditions for at least two generations before use in lifespan analysis. Statview 5.01 (SAS, Cary, North Carolina, United States) software was used for statistical analysis and to determine means and percentiles. In all cases, *p* values were calculated using the Log-rank (Mantel-Cox) method.

#### Dauer assays*.*

For RNAi experiments, *daf-2(e1370)* animals were cultured at 20 °C on plates seeded with various RNAi clones or vector control, and their F1 eggs were transferred to 22.5 °C. Following incubation for four days, the number of dauers was determined using a dissecting microscope. 30–50% of the animals on vector control became dauers at 22.5 °C. Approximately 50–100 animals were scored in each experiment. *daf-2(e1370)* worms grown on *ddl-3* RNAi gave rise to almost no progeny; therefore, this RNAi clone was not assayed.

For addressing whether germline ablation affects dauer formation, the number of dauers induced by either *daf-2(e1370)* or *daf-2(e1370); mes-1(bn7)* at 22.5 °C was assayed*.* This *mes-1* allele causes ~50% of animals to lack germ cells and live long [55]: 61 ± 20% of *daf-2(e1370)* mutants formed dauers (*n* = 204) and 57 ± 13% of *daf-2*(*e1370); mes-1(bn7)* double mutants (*n* = 267) formed dauers (*p =* 0.42). (The sterile and fertile *daf-2; mes-1* double mutants formed dauers at equal frequency at 22.5 °C.)

#### Stress response assays.

For the thermotolerance analysis, synchronized N2 animals grown on control or RNAi bacteria were shifted to 35 °C as 3-d-old adults; for the oxidative stress analysis, synchronized animals were exposed to 300 mM paraquat (Sigma, St. Louis, Missouri, United States) as 5-d-old adults. Survival was scored every 2 to 3 h after the treatment. Statview 5.01 (SAS) software was used for statistical analysis and to determine means. In all cases, *p* values were calculated using the Log-rank (Mantel-Cox) method.

#### Progeny production assays.

N2 eggs were incubated at 20 °C on plates seeded with various RNAi clones, and 24 late L4 stage worms were picked for each treatment and transferred to fresh RNAi plates every 12 h for 4–5 d. After this period, the worms were transferred every 24 h. Worms that crawled off the plates, bagged, or exploded were censored. All plates were then incubated at 20 °C for about 2 d and shifted to 4 °C. The number of worms that developed was determined at the end of the experiment.

#### Quantitative RT-PCR analysis.

Total RNA was isolated from approximately 5,000 d 1 adult worms and cDNA was made from 4 μg of RNA using Superscript II RT (Invitrogen, Carlsbad, California, United States). *eat-2(ad1116)* animals were harvested 8–18 h after N2, due to their delayed development. TaqMan real-time qPCR experiments were then performed by the Biomolecular Resource Center at UCSF as described in the manual using ABI Prism7900HT (Applied Biosystems, Foster City, California, United States). Primers and probes designed specifically for *act-1,* *sams-1, rab-10,* *drr-1,* and *drr-2* are listed below.

Primers:

Act-1–720F: 5′-CTACGAACTTCCTGACGGACAAG-3′

Act-1–821R: 5′-CCGGCGGACTCCATACC-3′

Sams-1–209F: 5′-TCCGTCGTGTCATCGAAAAG-3′

Sams-1–275R: 5′-TTGCAGGTCTTGTGGTCGAA-3′

Rab-10–514F: 5′-GCTAAGATGCCTGATACCACTGA-3′

Rab-10–585R: 5′-ACTCTGCCTCTGTGGTTGCA-3′

Drr-1–137F: 5′-GGATTCTTTGGTTTACTCTAATTGTTCA-3′

Drr-1–208R: 5′-TCCGCAGGGCGAAGATT-3′

Drr-2–530F: 5′-TGAAGCCCCGTACCACAGA-3′

Drr-2–596R: 5′-CTTGGTCTCCTCTTCTTCTTGCT-3′

Probes (All probes listed here were labeled with FAM at the 5′ end and Black hole Quencher at the 3′ end):

Act-1-T: 5′-AAACGAACGTTTCCGTTGCCCAGAGGCTAT-3′

Sams-1–230T: 5′-TTGGATTCACCGACTCCAGCATTGG-3′

Rab-10–541T: 5′-CAATCCCGCGATACGGTGAATCCA-3′

Drr-1–166T: 5′-TTAATTATTTCCGCGGCGGCAACG-3′

Drr-2–551T: 5′-CGCTGAGATCGAGGCTCGCAA-3′

## Supporting Information

Figure S1 Summary of RNAi Longevity Screen (45 KB PPT)

Figure S2 Comparison of *daf-2* RNAi Clones (72 KB PPT)

Table S1 Complete Lifespan Analysis Data of RNAi Clones That Extend Lifespan (839 KB DOC)

Table S2 Lifespan Analysis of Library Clones Encoding Known (Non-Neuronal) Longevity Genes (48 KB DOC)

Table S3 Pumping Rate and Body Length of N2 Animals Grown on Mitochondrial RNAi Clones Found at DOI: 10.1371/journal.pgen.0010017.st003 (49 KB DOC)

## Abbreviations

DR: dietary restriction
RNAi: RNA interference
TPR: tetratrico-peptide-repeat
