# Supplementary material for: New Genes Tied to Endocrine, Metabolic, and Dietary Regulation of Lifespan from a Caenorhabditis elegans Genomic RNAi Screen
Source: PLoS Genet. 2005 Jul 25;1(1):e17. doi: 10.1371/journal.pgen.0010017 (PMC1183531; doi:10.1371/journal.pgen.0010017)
Supplement: Figure S1 — (45 KB PPT) [file pgen.0010017.sg001.ppt]

## Slide 1
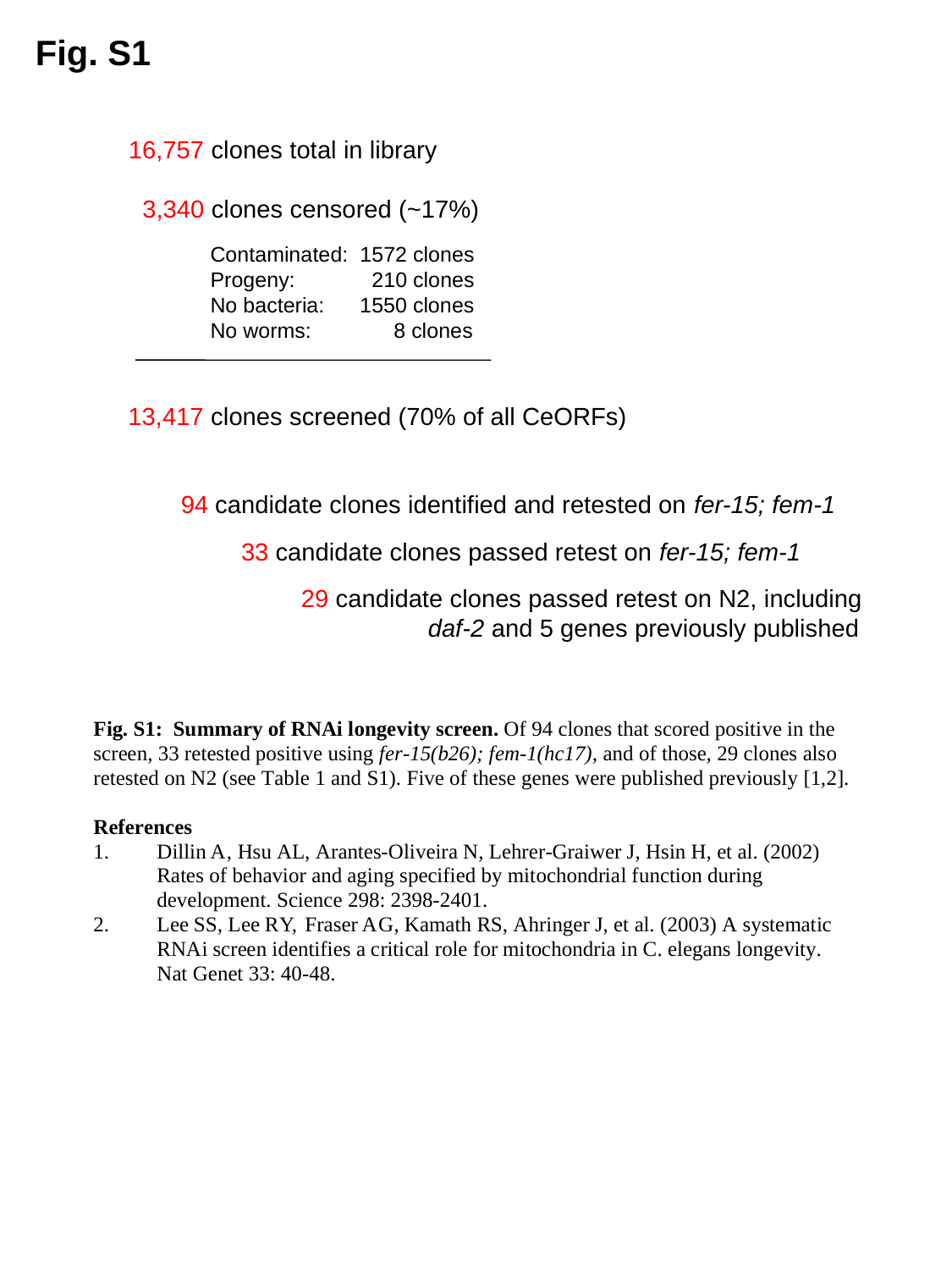

# Fig. S1
16,757 clones total in library
 3,340 clones censored (~17%)
13,417 clones screened (70% of all CeORFs)
94 candidate clones identified and retested on fer-15; fem-1
33 candidate clones passed retest on fer-15; fem-1
29 candidate clones passed retest on N2, including
	daf-2 and 5 genes previously published
	Contaminated: 1572 clones
	Progeny: 210 clones
	No bacteria: 1550 clones
	No worms: 8 clones
