# Supplementary figures and images for: New Genes Tied to Endocrine, Metabolic, and Dietary Regulation of Lifespan from a Caenorhabditis elegans Genomic RNAi Screen
Source: PLoS Genet. 2005 Jul 25;1(1):e17. doi: 10.1371/journal.pgen.0010017 (PMC1183531; doi:10.1371/journal.pgen.0010017)

## Slide 1
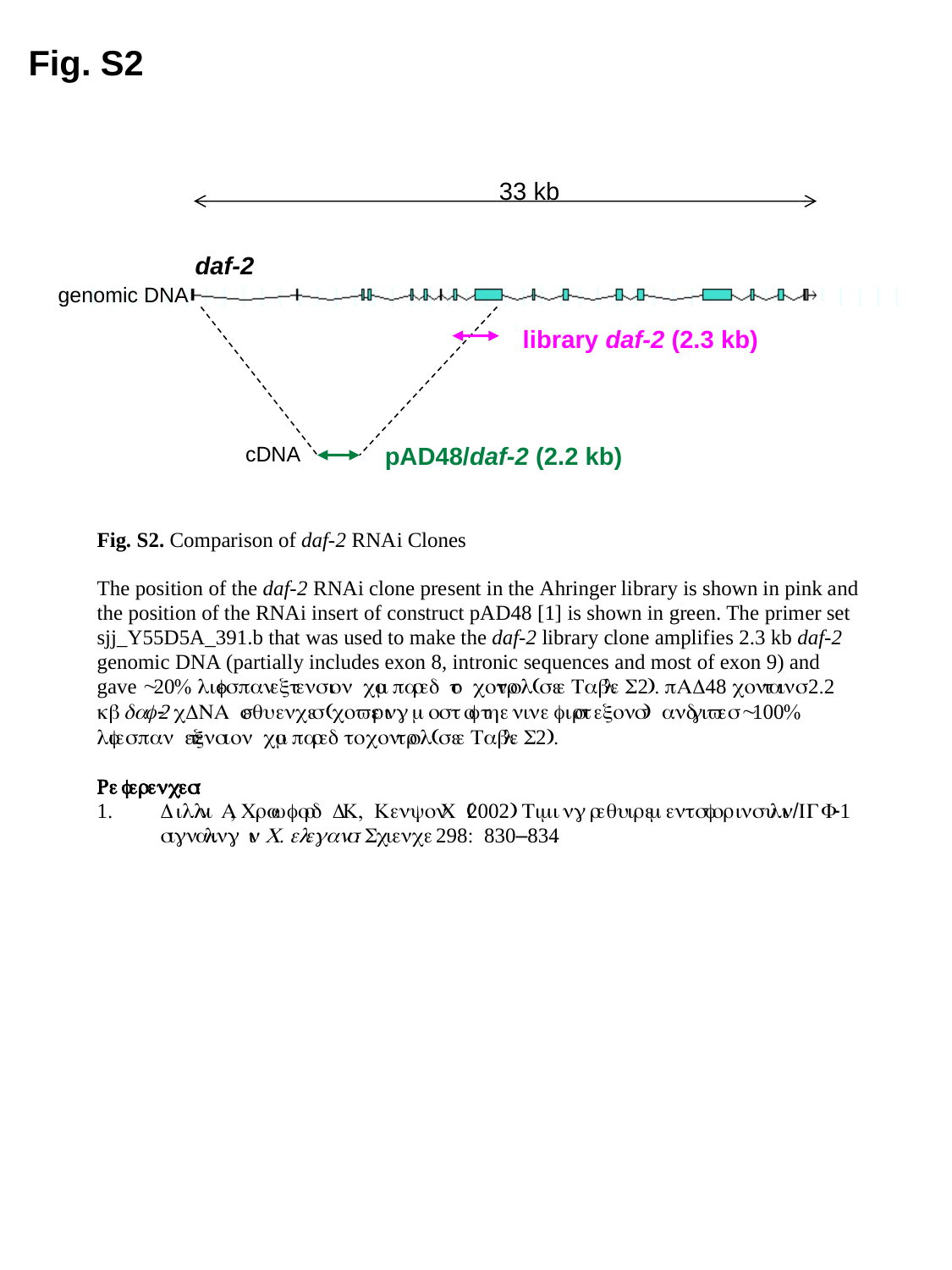

# Fig. S2
33 kb
daf-2
genomic DNA
library daf-2 (2.3 kb)
cDNA
pAD48/daf-2 (2.2 kb)

Supplement: Figure S2 — (72 KB PPT) [file pgen.0010017.sg002.ppt]
