# Supplementary material for: New Genes Tied to Endocrine, Metabolic, and Dietary Regulation of Lifespan from a Caenorhabditis elegans Genomic RNAi Screen
Source: PLoS Genet. 2005 Jul 25;1(1):e17. doi: 10.1371/journal.pgen.0010017 (PMC1183531; doi:10.1371/journal.pgen.0010017)
Supplement: Table S1 — (839 KB DOC) [file pgen.0010017.st001.doc]

**Table S1.** Complete Lifespan Analysis Data of RNAi Clones That Extend Lifespan

**Table S1.** Complete Lifespan Analysis Data of RNAi Clones That Extend Lifespan (continued)

**Table S1.** Complete Lifespan Analysis Data of RNAi Clones That Extend Lifespan (continued)

**Table S1.** Complete Lifespan Analysis Data of RNAi Clones That Extend Lifespan (continued)

**Table S1.** Complete Lifespan Analysis Data of RNAi Clones That Extend Lifespan (continued)

**Table S1.** Complete Lifespan Analysis Data of RNAi Clones That Extend Lifespan (continued)

**Table S1**. ‘Control’, mean adult lifespan, in days, of animals grown on control bacteria (no RNAi insert). ‘RNAi’, mean adult lifespan, in days, of animals grown on specific RNAi clone. ‘75%’ is the mean lifespan, in days, of the 75^th^ percentile (the age at which the fraction of animals alive reaches 0.25). *p*-values were calculated by pair-wise comparisons to the control (no RNAi insert). We used Statview 5.01 (SAS) software for statistical analysis and to determine means and percentiles. The Log-rank (Mantel-Cox) test was used to test the hypothesis that the survival functions between two different treatments (RNAi clone and control bacteria) were equal. ‘n’, shows number of observed deaths relative to total number of animals started on RNAi treatment. The difference between these numbers represents the number of animals censored during the experiment, and includes animals that exploded, bagged (ie, exhibited internal progeny hatching), or crawled off the plates. 'a‘, lifespan extension of animals grown on RNAi clone compared to control bacteria (no RNAi insert). Animals grown on control or experimental RNAi plates were cultured in parallel and transferred to new plates approximately every week at the same time (see Methods). Repetitions of the same experiments are listed in order. 'TRP', Tetratrico-peptide-repeats. ‘#‘, *eat-2* animals raised on *pat-4* or *pat-6* RNAi appeared unhealthy. Lifespan data on *cyc-1*, *cco-1*, *nuo-2*, and *atp-3* was published previously [1]. The genes *cchl-1* and *cco-1* were previously identified in the Ruvkun lab’s Chromosome I/II screen [2]. Strains employed were *fer-15(b26); fem-1(hc17)*, *daf-16(mu86)*, *daf-2(e1370)*, *glp-1(e2141)*, *daf-12(rh41rh411),* and *eat-2(ad1116)*. Lifespan experiments were carried out at 20 °C (that is, *fer-15(b26); fem-1(hc17)* and *glp-1(e2141)* eggs were incubated at 25 °C until adulthood and lifespan analysis of adult animals was performed at 20 °C) except for experiments marked ‘^’, which were carried out at 25°C.

**References**

1. Dillin A, Hsu AL, Arantes-Oliveira N, Lehrer-Graiwer J, Hsin H, et al. (2002) Rates of behavior and aging specified by mitochondrial function during development. Science 298: 2398-2401.

2. Lee SS, Lee RY, Fraser AG, Kamath RS, Ahringer J, et al. (2003) A systematic RNAi screen identifies a critical role for mitochondria in C. elegans longevity. Nat Genet 33: 40-48.
