# Supplementary material for: New Genes Tied to Endocrine, Metabolic, and Dietary Regulation of Lifespan from a Caenorhabditis elegans Genomic RNAi Screen
Source: PLoS Genet. 2005 Jul 25;1(1):e17. doi: 10.1371/journal.pgen.0010017 (PMC1183531; doi:10.1371/journal.pgen.0010017)
Supplement: Table S2 — (48 KB DOC) [file pgen.0010017.st002.doc]

#### Table S2. Lifespan Analysis of Library Clones Encoding Known (Non-Neuronal) Longevity Genes

**Table S2**. Mean lifespan extensions of RNAi-treated *fer-15(b26); fem-1(hc17)* mutants. ‘a’, lifespan extension of *fer-15(b26); fem-1(hc17)* animals grown on RNAi clones relative to worms grown on control bacteria (no RNAi insert). *p*-values were calculated by pair-wise comparisons to control of the experiment. ‘n’, shows number of observed deaths relative to total number of animals started on RNAi treatment. The difference between these numbers shows the censoring rate of the experiment and includes explosion, bagging and missing worms. ‘pAD48/*daf-2*’, *daf-2* clone previously described [1]. ‘*daf-2*’ is clone from library amplified by primer set sjj_Y55D5A_391.b. ‘No bacteria’, bacterial clone was annotated in library database to exhibit no growth. ‘No growth’, bacterial clone was expected to grow but showed no growth when inoculated from our library copy. Data shows result of one lifespan trial, however, *daf-2* and pAD48/*daf-2* were tested multiple times with similar results (see Table S1 and data not shown). *fer-15(b26); fem-1(hc17)* eggs were incubated at 25 °C until adulthood and lifespan analysis of adult animals was performed at 20 °C.

**Reference:**

1. Dillin A, Crawford DK, Kenyon C (2002) Timing requirements for insulin/IGF-1 signaling in *C. elegans.* Science 298: 830–834.
