# Supplementary material for: New Genes Tied to Endocrine, Metabolic, and Dietary Regulation of Lifespan from a Caenorhabditis elegans Genomic RNAi Screen
Source: PLoS Genet. 2005 Jul 25;1(1):e17. doi: 10.1371/journal.pgen.0010017 (PMC1183531; doi:10.1371/journal.pgen.0010017)
Supplement: Table S3 — Found at DOI: 10.1371/journal.pgen.0010017.st003 (49 KB DOC) [file pgen.0010017.st003.doc]

Table S3. Pumping Rate and Body Length of N2 Animals Grown on Mitochondrial RNAi Clones

**Table S3**. Mean length and mean pumping rate were measured on 8-20 N2 animals grown at 20 °C on d 1 or 2 of adulthood, respectively. All RNAi clones (except *nuo-5*) were assayed in one experiment including Control (a). Control (b) served as a control for the *nuo-5* experiment. Similar results were obtained for *fer-15; fem-15* animals assayed on respiratory-chain RNAi clones (data not shown). ‘SD’, standard deviation, ‘ND’, not determined, ‘*’, *isp-1(qm150)* is a mutant defective in a component of Complex II.
